# ILC2 Orchestration of Local Immune Function in Adipose Tissue

**DOI:** 10.3389/fimmu.2019.00171

**Published:** 2019-02-07

**Authors:** Cécile Bénézech, Lucy Helen Jackson-Jones

**Affiliations:** ^1^BHF Centre for Cardiovascular Science, University of Edinburgh, Edinburgh, United Kingdom; ^2^Division of Biomedical and Life Sciences, Faculty of Health and Medicine, Lancaster University, Lancaster, United Kingdom

**Keywords:** adipose, ILC2, FALCs, innate, antibodies, thermogenesis, mucosal, atherosclerosis

## Abstract

ILC2s were originally identified as IL-5 and IL-13 secreting “natural helper cells” present within the fat-associated lymphoid clusters of the mesenteries in both mouse and man. The presence of ILCs in adipose tissue has more recently expanded to include all ILC groups. Since their initial discovery, our knowledge of these cells and their role in adipose immune responses has expanded significantly. In this review we summarize the current literature on the role that ILC2s play in orchestrating adipose tissue function in both lean and obese states. We go on to address new data detailing interactions of adipose ILCs with innate like B-cells (IBC) and discuss how this interaction results in localized protection of mucosal sites during infection and inflammation via the production of innate antibodies.

## Introduction

Innate lymphoid cells are the newest kids on the block in terms of innate immune cell function, however the previous 8 years have revealed a wealth of information on these previously enigmatic lymphocyte like cells. In a non-activated state, ILCs possess lymphocyte morphology but lack the expression of surface markers used to define other immune cell populations. ILCs are thus described as “lineage negative.” Non-cytotoxic ILCs are currently segregated into three transcriptionally defined groups that mirror the four major T-helper cell subsets. Tbet dependent ILC1s which secrete IFNγ and TNFα, GATA3 dependent ILC2s which secrete IL-5/IL-13 [and can secrete IL-10 (1)], RORγt dependent ILC3s which secrete IL-17A/IL-22, and include a population of Lymphoid tissue inducer (LTi) cells which are critical for secondary lymphoid organ development (2) and finally, Id3 dependent ILCregs which produce IL-10 and require autocrine TGF-β1 (3). In addition to these non-cytotoxic cell types, are the classical cytotoxic NK cells that are important for protection against viruses and cancer. Although ILCs were first described as natural helper cells (ILC2) in the fat-associated lymphoid clusters (FALCs) of the mesenteries where they support antibody responses, their presence and importance has since been extended to the whole adipose organ with ILCs having been reported in most fat depots. ILCs are now considered as key regulators of adipose tissue function. IBCs are B cells with “innate like” properties; they have a poly-specific B-cell receptor repertoire and rapidly produce polyclonal IgM in response to both self and microbial antigens (4). Here we will discuss (1) the central regulatory role of ILC2 in the regulation of adipose tissue homeostasis and (2) the key role of ILCs in activation of the IBC compartment during infection at mucosal sites.

## ILC2s are Critical Regulators of Type 2 Immune Cells to Maintain White Adipose Tissue Homeostasis

Recently, it has become apparent that type 2 immune cells play a critical role in the maintenance of homeostasis in lean, healthy adipose tissue and that ILC2 are central regulators of this function. Type 2 immune cells including ILC2, T regulatory cells (Treg), T-helper type 2 cells (Th2), Eosinophils, mast cells, and M2 macrophages are prevalent in healthy adipose tissue where they contribute to adipose tissue remodeling, counteracting the inflammatory effect of obesity and inducing browning of white adipose tissue (5, 6). Here, we will concentrate on the role of ILC2 in orchestrating the function of type 2 immune cells in adipose tissue.

### ILC2s and Immune Homeostasis in White Adipose Tissue

**Figure 1:**
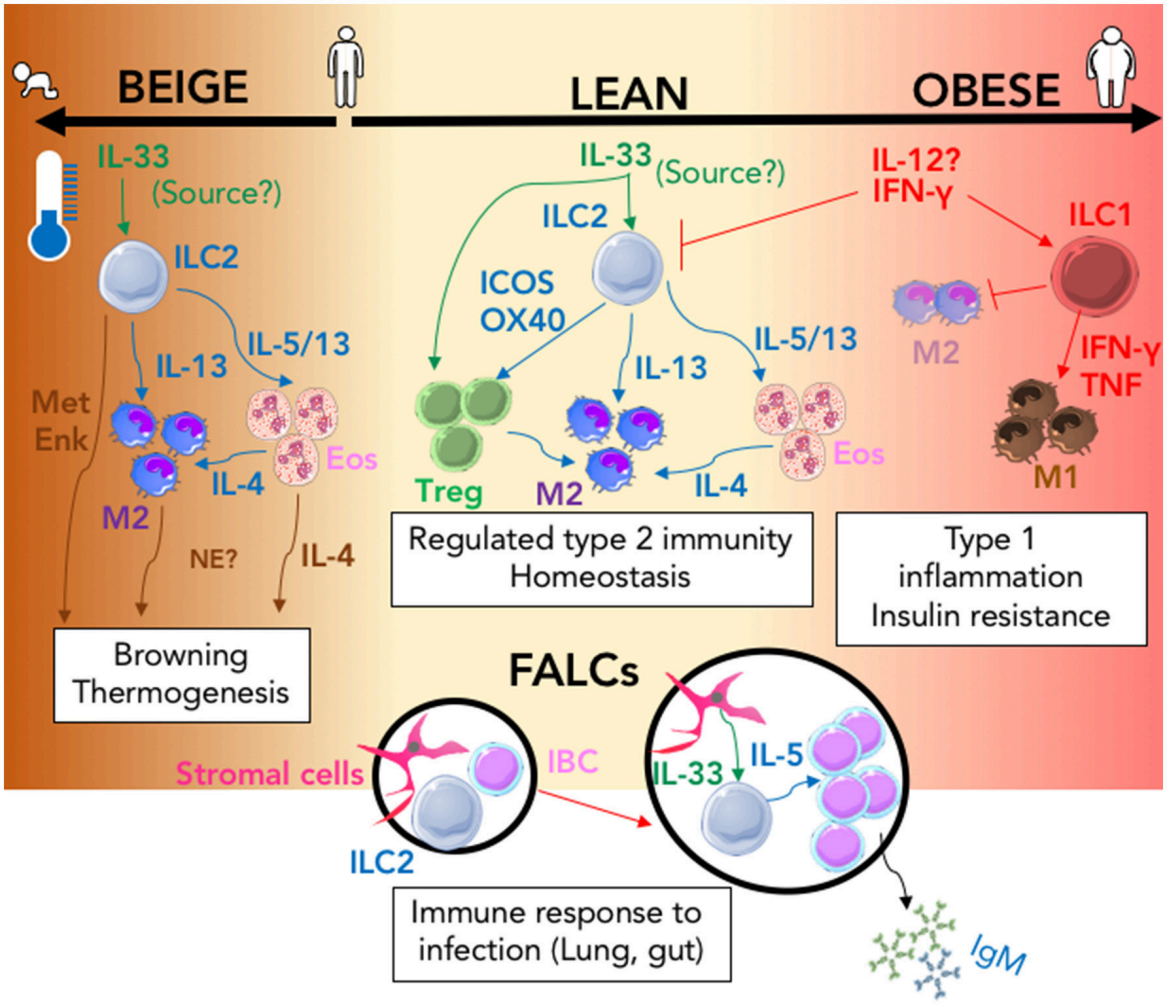
The ILC2 driven interactions that regulate immune adipose function. In the lean state (center; cream) IL-33 action (green arrows) signals to both T-regulatory cells (Treg) and ILC2 resulting in regulated Type-2 immunity via the activity of secreted and membrane bound type-2 signals (blue arrows); this response is amplified in the presence of lower ambient living temperature and during infancy and can result in browning thermogenesis within adipose tissue (Left; brown). Type-2 signals that can control browning are shown (brown arrows). In the obese state (right; pink) Inflammation mediated by type-1 signals (red arrows) promotes the activation of ILC1 and the inhibition of ILC2 which results in inhibition of M2 and expansion of the M1 macrophage population which contribute to the development of insulin resistance. During Type-2 inflammation within the lung or gut, ILC2 containing FALCs (Black circles) expand; IL-33 produced by stromal cells (green arrow) increases IL-5 secretion (blue arrow) from ILC2 which induces innate like B cell (IBC) proliferation and secretion of IgM. (MetEnk, methionine-enkephalin peptides; NE, norepinephrine; Eos, Eosinophils; IBC, Innate Like B cell; M1/M2, M1, or M2 macrophage; FALCs, Fat-associated lymphoid clusters).

ILC2s are present within visceral adipose tissue (VAT), where they are the predominant producers of IL-5 and IL-13 at homeostasis and following prolonged exposure to IL-33 or helminth infection (7, 8). Th2 cells remain a minor population of IL-5 and IL-13 producing cells within the VAT even during helminth infection (8). In lean adipose tissue, IL-33 drives the recruitment and/or proliferation of ILC2 but the cellular origin of IL-33 and the mechanisms leading to its secretion at homeostasis remains poorly understood. While we reported that Gp38^+^ stromal cells of fat-associated lymphoid clusters express high levels of IL-33, others showed that IL-33 is also expressed by Gp38^+^ fibroblasts, Cadherin-11^+^ mesenchymal cells, or endothelial cells of the stromal vascular fraction of adipose tissue (9–12). It is likely that the relevant source of IL-33 in adipose tissue is context dependent and further work is needed to elucidate the mechanism of IL-33 action in adipose tissue. Tissue ILC2s are key producers of systemic IL-5 required for homeostatic eosinophil maintenance (13). In adipose tissue, secretion of IL-5 by ILC2 is essential for the recruitment and maintenance of eosinophils (8) and is dependent on IL-33 (8) (Figure 1). Secretion of IL-13 and IL-4 by ILC2 and eosinophils is critical for the maintenance of alternatively activated or M2-like adipose tissue macrophages and glucose homeostasis (8, 14). The precise phenotype and origin of these macrophages is not known. Interestingly IL-33 has been shown to be competent to induce macrophage proliferation independently of IL-4Rα expression in other non-adipose macrophages populations (15) and whether IL-33 can directly activate adipose tissue macrophages remains to be investigated.

Pioneering work by the group of Diane Mathis demonstrated the existence of a unique subset of GATA-3^+^ PPARγ^+^ regulatory T cells in adipose tissue important for preventing insulin resistance (16, 17). Regulatory T cells in adipose tissue express the IL-33 receptor ST2 and require IL-33 for their maintenance (18). Additionally, expression of ICOSL by adipose tissue ILC2 provides additional signaling through ICOS in regulatory T cells for their accumulation within VAT (10). Halim et al. elegantly advance these finding by showing that in the absence of ILC2s or specifically the absence of OX40L expression by ILC2s there is a significant deficit in the number of GATA3^+^ T-regulatory cells within the perigonadal adipose tissue following IL-33 delivery (19).

### ILC2s and Adipose Tissue Browning

Brown and beige adipose tissue are fat depots specialized in the dissipation of energy for the production of heat. While brown adipose tissue is mostly found in infants and regresses with age, white adipose tissue can undergo “browning” to form beige adipose tissue, expressing the thermogenic protein Ucp1 during exposure to cold (20). Two distinct mechanisms involving ILC2s have been implicated in the browning of adipose tissue. Mechanism one relies on the IL-33 dependent induction of methionine-enkephalin peptide release from ILC2s that acts directly on adipocytes to upregulate UCP-1 and induce beiging (21). The second published mechanism involves pharmacologic expansion and activation of ILC2 with IL-33 in thermoneutral mice which induces the proliferation of adipocytes and their differentiation into beige adipocytes (22). This is dependent on the release of IL-4 and IL-13 by ILC2 and the direct activation of adipocyte precursor cells via the IL-4Rα (22). ILC2 may also be important for the activation of eosinophils during acute cold exposure and the secretion of IL-4/13, which have been reported to induce browning through activation of alternatively activated macrophage production of catecholamines (23). However, the mechanisms leading to secretion of IL-33 upon cold exposure were not elucidated. The production of catecholamines by alternatively activated macrophages is controversial with a recent report stating that alternatively activated macrophages do not produce catecholamines and are thus unlikely to have a direct role in adipocyte metabolism or adaptive thermogenesis (24).

#### Is There a Link Between the Gut Mucosa and the Metabolic Regulatory Function of ILC2 in Adipose Tissue?

In the small intestine, the release of IL-5 and IL-13 by ILC2 is increased by food intake, leading to fluctuation in the levels of circulating eosinophils during the day (13). It would be interesting to know if the secretion of IL-5 and IL-13 or other important mediators such as methionine-enkephalin peptides by adipose tissue ILC2s fluctuates with food intake, thus allowing the synchronization of adipose tissue function with food intake via immune regulation.

## A Link Between Adipose Tissue ILC2s and Metabolic Dysfunction

During obesity the number of ILC2s decreases in adipose tissue both in mouse and human, leading to decrease in overall Type-2 immunity and increased inflammation in adipose tissue. Importantly, the loss of ILC2 in obesity can be reversed by IL-33 injection in obese mice restoring glucose tolerance and insulin sensitivity. However, the mechanisms leading to the loss of ILC2 during obesity are not well-understood. Interestingly, a population of ILC1s expand in the adipose tissue during diet-induced obesity and produce IFN-γ in response to IL-12, contributing to inflammation and insulin resistance (25). IFN-γ has an antagonistic effect on ILC2 (10) which may be responsible for the loss of ILC2 during obesity. It is also possible that IFNγ and or IL-12 drives the conversion of ILC2 toward ILC1 during diet-induced obesity, as described in response to IL-12 (26). In addition, upregulation of PD-1 expression on ILC2 and its engagement via PD-L1^hi^ M1 macrophages has recently been described to inhibit the protective function of ILC2s during obesity. Within obese adipose, increased PD-1 expression on ILC2s was dependent on TNFα and IL-33 (27).

In the second half of this mini-review, the original role of ILCs in the initiation of local immune function in FALCs is discussed and extended to include the newly described pleural FALCs (11, 28, 29); finally we discuss the interaction between ILC2s, IBCs, and IgM during atherosclerosis.

## Fat-Associated Lymphoid Cluster Function in Mucosal Defense

**Figure 2:**
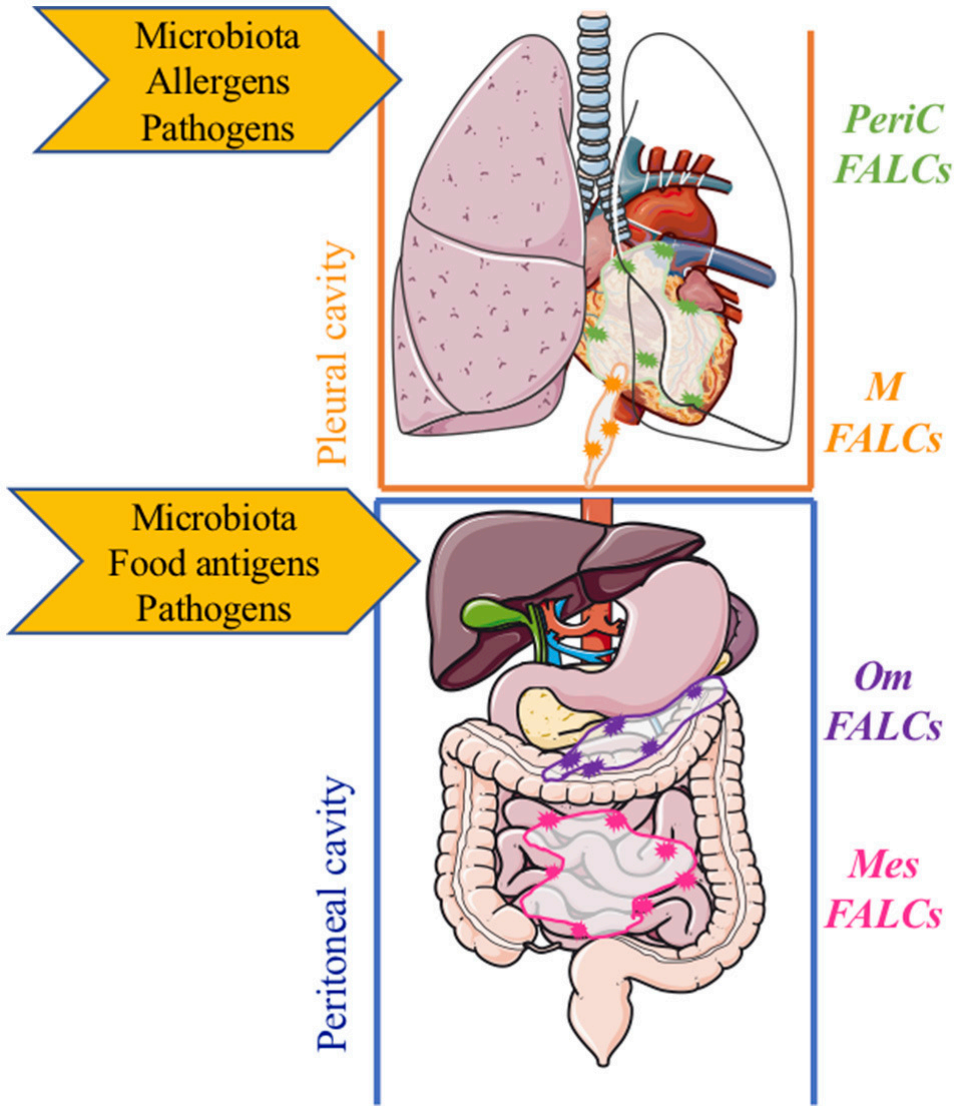
Compartmentalized protection of mucosal sites by fat-associated lymphoid clusters within body cavities. Within the pleural cavity, protection from/regulation of, microbiota, infection, inflammation, and damage is mediated by inducible FALCs within the pericardium (green) and mediastinum (orange). Within the peritoneal cavity, protection from/regulation of microbiota, infection, inflammation, and damage is mediated by FALCs within the omentum (purple) and mesenteries (pink) m, mediastinal; PeriC, pericardial; om, omental; mes, mesenteric; FALCs, Fat-associated lymphoid clusters.

The peritoneal and pleural cavities, primarily considered as sites of macrophage (30) and B1 cell residence represent compartments that demarcate, contain and protect the boundaries between three major mucosal sites directly exposed to environmental antigens; namely the lungs, the intestines and the reproductive tract (of females) (Figure 2). Immune protection within the body cavities is co-ordinated by small, inducible lymphoid clusters found within specialized small adipose tissues (the mediastinum, pericardium, mesenteries, and omentum). Initially described as “milky-spots” within the omentum (31) these inducible structures were rebranded in 2010 as Fat-Associated Lymphoid Clusters or FALCs (32). FALCs are local hubs that are important for providing a second line of defense between the mucosal surfaces and a systemic immune response, working to compartmentalize antibody mediated immune responses within body cavities. Evidence supporting FALC orchestration of antibody responses within the body cavities is mounting, with multiple reports linking FALCs to the initiation of T-independent and T-dependent immune responses (11, 29, 32–34).

## FALCs, ILCs, and the Initiation of Innate Like B Cell Responses

### Intestinal Barrier Functions

FALCs were identified as immune cell aggregates within the mesenteries, that were enriched in lineage negative, c-Kit^+^, Sca-1^+^ cells; these cells are now known as ILC2s (32, 35, 36). ILC2s are potent producers of IL-5 and IL-13; detectable levels of both cytokines are induced in the peritoneal lavage of *Rag2*^−/−^ mice which do not have mature T or B cells, but are absent from γ*c*^−/−^*Rag2*^−/−^ following infection with the tissue migrating parasite *Nippostrongylus brasiliensis* (32). This result highlighted the potency of common-gamma chain receptor dependent innate immune cells for the initiation of immune responses within the peritoneal cavity in the context of intestinal worm infection. IL-5 is a critical growth factor for B1 B cells (37); Moro and colleagues showed, using elegant *in vivo* transfers and *in vitro* co-cultures of ILC2 with peritoneal B-cells in the presence or absence of a blocking antibody against IL-5, that ILC2s provide support for B1 cell self-renewal (32). ILC2s isolated from mesenteric FALCs were also shown to be competent for the induction of IgA secretion by peritoneal B cells *in vitro* (32). Peritoneal B1 cells have been shown to migrate to the intestinal lamina propria in order to secrete IgA (38, 39). In addition to the conventional “Type-2” cytokines described above, ILC2 have also been shown to secrete IL-6 (40, 41). As IL-6 has been described to induce antibody production by B-cells, as well as act as a growth factor for plasmablasts (42) and contribute to the regulation of T follicular helper cells (43), it is plausible that ILC2 secretion of this cytokine locally modifies FALC B-cell function; a hypothesis that warrants further experimental investigation to confirm. Contrary to secondary organs, the development of FALCs is not dependent on ILC3 as shown by the normal development and composition of FALCs in *Rorc*^−/−^ mice (29). However, studies in germ free mice revealed that the number of FALCs forming in the mesenteries is decreased indicating that factors derived from the commensal flora are important to drive the formation of FALCs. ILC3s are an important innate source of GM-CSF, a cytokine required for the induction of IgM by innate response activator (IRA) B cells (44). Competency to support IgA secretion by B1 was also reported for peritoneal macrophages, which had been exposed to omentum culture supernatant (45). Given the almost certain presence of ILC derived factors within the omental culture supernatant, it is hard to know what component of the IgA secretion mediated by peritoneal macrophages is in part dependent upon ILCs. A thorough characterization of the ILC occupation of the murine omentum has not been carried out; however a recent report characterized the presence of ILCs in multiple human tissues including detailing the presence of ILC1 like cells within the omentum (46).

#### Pulmonary Barrier Functions

IgM is a large antibody and as such secretion of IgM into the circulation does not guarantee its presence at tissue sites where it is required. In the global absence of the IL-33R ST2, the secretion of IgM from FALCs within the pleural cavity is ablated (11). This is not a direct effect on the B-cells as co-transfer of IL-33R sufficient and deficient B-cells resulted in comparable induction of B-cell activation following *Alternaria alternata* delivery. Utilizing blocking antibodies against IL-5 delivered directly into the pleural space, we concluded that the IL-33 was acting via an IL-5 producing intermediate population of cells. ILC2s were the only cells found to be expressing IL-5 within FALCs of the pleural cavity during type-2 inflammation (11). Thus, the presence of IgM secreting B-cells within FALCs in the context of type-2 inflammation is assumed to depend upon IL-5 secretion from IL-33 activated ILC2s. The link between ILC2 and antibody production within the thoracic cavity was also made by Drake et al. (47) who showed that *in vitro* culture of lung derived ILCs with splenic B cells resulted in antibody production (47). However, as there are fewer B-cells within the lungs and because fluid phase B cells isolated from the pleural space do no secrete antibodies, it is likely that pleural FALCs are the sites where the ILC/B cell interactions take place in the thoracic cavity. In support of a tight immune crosstalk between lung and pleural space is a report showing that delivery of GM-CSF secreting IRA B cells into the pleural space mediates protection from pneumonia (48). Neither the role of FALCs in the activation of the transferred IRA B cells nor the requirement for lung or FALC resident ILCs in this process was investigated. This study serves to further highlights the crosstalk which occurs between mucosal tissues and their associated serous cavities.

#### Is FALC Derived IgM Atheroprotective?

Innate like B-cells (IBCs) can be both protective and pathogenic in atherosclerosis. Recognition of oxidation specific epitopes on low density lipoproteins (LDL) (49) by natural IgM plays a protective role in atherosclerosis and clinical studies show that lower levels of IgM correlates with increased risk of cardiovascular diseases. The production of atheroprotective IgM by IBCs is dependent on IL-33 (50), IL-5 and IL-5 producing ILC2 (51, 52), a signaling loop that is active in FALCs (11). Importantly, it has been shown that the number of FALCs in the para-aortic adipose of ApoE^−/−^ mice increases in the vicinity of atherosclerotic lesions (52) and that they contain IBC producing atheroprotective IgM (53). This suggests that ILC2 regulation of local IgM secretion by FALC IBCs could be key to IBC mediated atheroprotection and that loss of ILC2 duringthe development of obesity could contribute to accelerated atherosclerosis.

## Summary

Since their initial discovery 8 years ago, ILC2s have emerged as major regulators of type-2 immunity in adipose tissue where they co-ordinate eosinophil, macrophage, adipocyte and IBC function. FALCs are specialized hubs that act as a second line of immune defense sitting behind the mucosal frontline. Key to the initiation of a FALC response is the local secretion of cytokines by FALC resident ILCs, which kick-start the ensuing immune response following detection of a danger signal (e.g., IL-33).

## Author Contributions

All authors listed have made a substantial, direct and intellectual contribution to the work, and approved it for publication.

### Conflict of Interest Statement

The authors declare that the research was conducted in the absence of any commercial or financial relationships that could be construed as a potential conflict of interest.
